# Differential Expression of Metallothionein Isoforms in Terrestrial Snail Embryos Reflects Early Life Stage Adaptation to Metal Stress

**DOI:** 10.1371/journal.pone.0116004

**Published:** 2015-02-23

**Authors:** Pierre-Emmanuel Baurand, Veronika Pedrini-Martha, Annette de Vaufleury, Michael Niederwanger, Nicolas Capelli, Renaud Scheifler, Reinhard Dallinger

**Affiliations:** 1 Chrono-Environnement, UMR 6249 University of Franche-Comté, 16 route de Gray, 25030, Besançon cedex, France; 2 Institute of Zoology, University of Innsbruck, Technikerstraße 25, A-6020, Innsbruck, Austria; 3 Department of Health Safety Environment, avenue des Rives du Lac, BP179, 70003, Vesoul cedex, France; Laboratoire de Biologie du Développement de Villefranche-sur-Mer, FRANCE

## Abstract

The aim of this study was to analyze the expression of three metallothionein (MT) isoform genes (*CdMT*, *CuMT* and *Cd/CuMT*), already known from adults, in the Early Life Stage (ELS) of *Cantareus aspersus*. This was accomplished by detection of the *MT* isoform-specific transcription adopting Polymerase Chain Reaction (PCR) amplification and quantitative Real Time (qRT)-PCR of the three *MT* genes. Freshly laid eggs were kept for 24 hours under control conditions or exposed to three cadmium (Cd) solutions of increasing concentration (5, 10, and 15 mg Cd/L). The transcription of the three *MT* isoform genes was detected via PCR in 1, 6 and 12-day-old control or Cd-exposed embryos. Moreover, the transcription of this isoform genes during development was followed by qRT-PCR in 6 and 12-day-old embryos. Our results showed that the *CdMT* and *Cd/CuMT* genes, but not the *CuMT* gene, are expressed in embryos at the first day of development. The transcription of the 3 *MT* genes in control embryos increased with development time, suggesting that the capacities of metal regulation and detoxification may have gradually increased throughout embryogenesis. However in control embryos, the most highly expressed *MT* gene was that of the *Cd/CuMT* isoform, whose transcription levels greatly exceeded those of the other two *MT* genes. This contrasts with the minor significance of this gene in adult snails and suggests that in embryos, this isoform may play a comparatively more important role in metal physiology compared to adult individuals. This function in adult snails appears not to be related to Cd detoxification. Instead, snail embryos responded to Cd exposure by over-expression of the *CdMT* gene in a concentration-dependent manner, whereas the expression of the *Cd/CuMT* gene remained unaffected. Moreover, our study demonstrates the ability of snail embryos to respond very early to Cd exposure by up-regulation of the *CdMT* gene.

## Introduction

Metallothioneins (MTs) constitute a superfamily of proteins that specifically bind to closed-shell metal ions (Zn^2+^, Cd^2+^, Cu^+^) via the sulfur atoms of their rich cysteine residues [1,2]. They are involved in metal-related tasks including metal ion detoxification and homeostasis [3–6], radical scavenging [7] and stress response [8]. Their specific physiological role may vary among animal species, the tissues in which they are expressed and the MT family to which they belong [9]. Terrestrial gastropods, for example, possess three MT isoforms, two of them with metal-selective features, performing metal-specific tasks [4]. Notably, one of these isoforms is cadmium (Cd)-selective and devoted to the detoxification of this harmful metal in digestive tissues [10]; the other is copper (Cu)-selective and involved in the regulation of Cu in connection with haemocyanin synthesis [11]. A third, non-specific MT isoform is expressed constitutively at only low basal levels and is hardly detected, if at all, in adult snails [12,13].

Terrestrial gastropods are particularly exposed to toxic metals in their habitat: not only do they move slowly and are in close contact with the soil substrate on which they thrive, but they also lay their eggs in the soil. Depending on meteorological and microclimatic conditions, the availability of trace elements to snails in the surrounding soil may fluctuate and change rapidly [14]. Perhaps as an adaptation to these alternating conditions, terrestrial gastropods are able to accumulate and detoxify certain toxic metals in their digestive tissues [10,15,16]. The non-essential Cd, in particular, is inactivated when bound to the expressed Cd-specific MT isoform [17,18]. The gene of this isoform is selectively up-regulated upon exposure to this metal, whereas the two other *MT* isoform genes do not respond to Cd or Cu exposure [13,19]. This Cd-specific response may confer to terrestrial helicids their relatively high tolerance for elevated environmental levels of this harmful metal.

The physiological and toxicological significance of the transcriptional regulation of the three *MT* isoform genes has recently been studied in adult garden snails, *Cantareus aspersus*, during a long-term metal exposure of 29 days [13]. It appears that the exclusive up-regulation of the *CdMT* isoform gene is in accordance with the relatively high Cd tolerance of this species, whereas the non-responsive transcription pattern of the two other *MT* genes indicates their comparatively low significance in the metabolic handling of Cd [13].

No data are available, however, on the presence and expression of the three MT isoforms in Early Life Stage (ELS) individuals (eggs, embryos) of *C*. *aspersus*. This may be important considering that in invertebrates, ELS may differ from adults in their sensitivity toward metal exposure [20,21]; the differential *MT* expression can play a crucial role in this concern [22]. Recent studies on *C*. *aspersus* embryos [23,24] reported a low sensitivity (based on hatching rate) of this species toward Cd with an EC_50_ of approximately 3.5 mg/L during continuous exposure over 20 days. Furthermore, very high Cd concentrations (up to 8 mg/L) are required to completely inhibit the hatching process. Baurand et al. [25] have shown genotoxic effects of Cd on *C*. *aspersus* embryos at exposure concentrations of 2 mg/L and a fragmentation of genomic DNA after 20 days of exposure to 6 mg/L Cd. The same Cd concentration disrupted the development of embryos by causing a decrease in cardiac rhythm and growth, and hatching delays [26]. Thus, overall, ELS individuals of *C*. *aspersus* exhibit a relatively high tolerance towards Cd exposure. However, little is known about the underlying mechanisms, particularly with respect to the expression of the three *MT* isoform genes and their potential role during Cd exposure.

Therefore, the aim of this study was to analyze the transcription of the three *MT* isoform genes in ELS of *C*. *aspersus* and to quantify their potential gene up-regulation due to Cd exposure by comparing Polymerase Chain Reaction (PCR) and quantitative Real Time PCR (qRT-PCR).

## Materials and Methods

### Exposure concentrations

Cd solutions were prepared by dissolving solid CdCl_2_ (99.99%, Sigma Chemical Co., St. Louis, MO; C-2544) in demineralized water (pH 6.2), which was also used as a control solution. The concentrations of Cd in solutions were measured by means of inductively coupled plasma atomic emission spectroscopy (ICP-AES) (ICAP 6000 series model radial, Thermo scientific, France). The quality of the results was verified using reference water (Hard Drink Water, ERM- CAO11a, Molsheim, France) that was Cd-certified at 4.94 μg/L (+/- 0.23 μg/L, average recovery of 93%). The nominal concentrations of exposure solutions were 5, 10 and 15 mg Cd/L, whilst actual concentrations were 3.8, 8.7, and 13.1 mg Cd/L, respectively.

### Exposure design and Cd treatment

**Fig 1:**
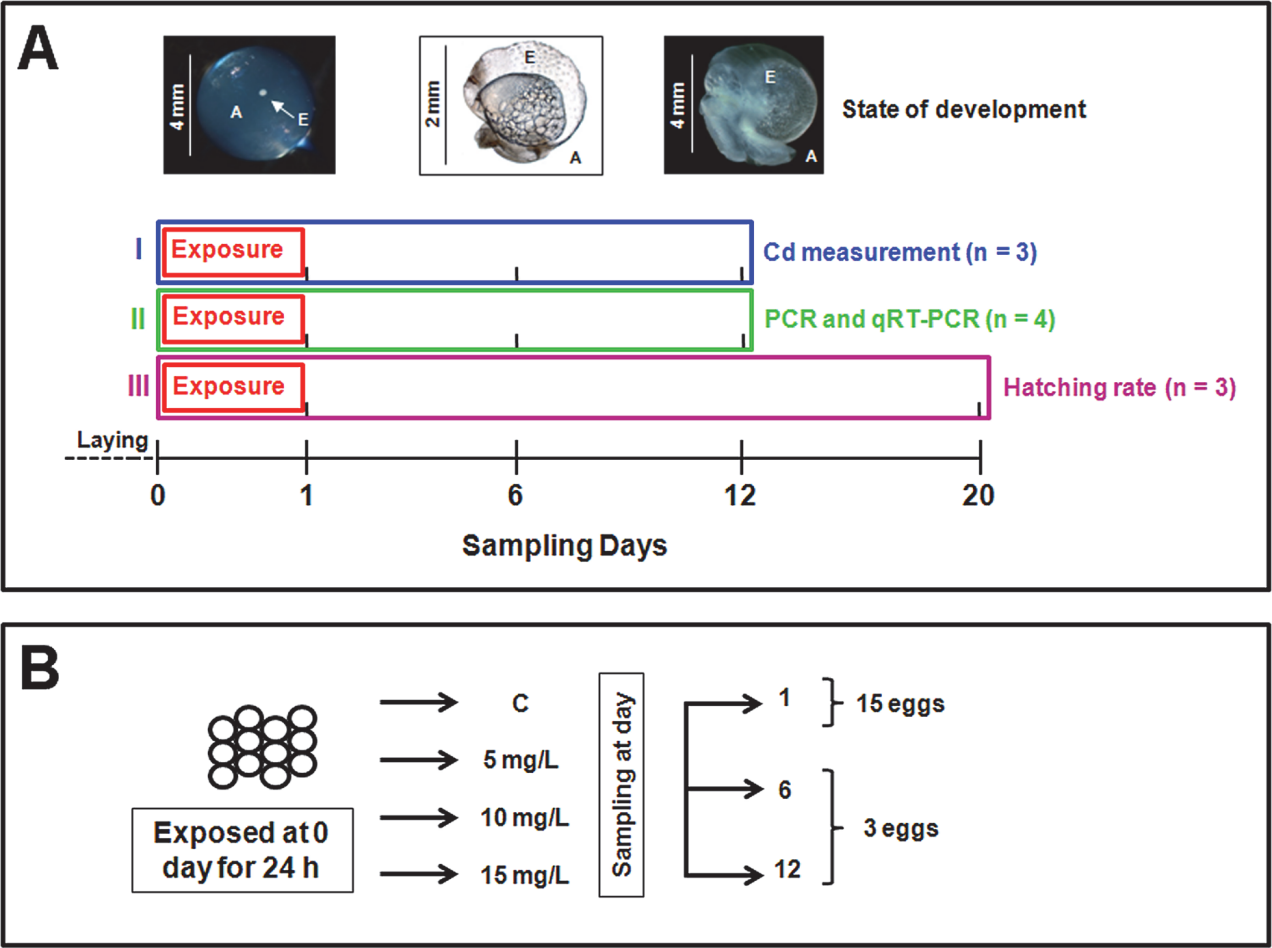
Scheme of exposure and sampling conditions during embryonic development of control and Cd-exposed eggs of *Cantareus aspersus* applied to the present study. (A) Illustration of developmental stages of snail eggs with specification of the albumen (A) and the embryo (E) (above), followed by details of exposure regimes and sampling dates applied for (I) the assessment of Cd concentrations in snail embryos, (II) RT and qRT-PCR detection of embryonic *MT* isoform genes, and (III) evaluation of successful hatching rates during development along the time axis (below), starting immediately after egg laying. n is the number of clutches used for each exposure regime. (B) Exposure scheme of eggs from each clutch (left-hand) treated with control solution (C) or with increasing nominal concentrations (5, 10 and 15 mg/L) of Cd; also shown are the numbers of eggs pooled (right-hand) to one sample at each of the days selected during the development.

Eggs were obtained from standardized laboratory rearing at the laboratory of Chrono-Environnement (University of Franche-Comté, Besançon, France) and were exposed using the liquid phase bioassay as previously described [23,24]. Briefly, clutches were separated into groups of 10 eggs each and placed into Petri dishes on four layers of paper (Quantitative filter paper grade 1 ashless, Whatman) dampened with 0.8 mL of demineralized water (control treatment) or one of the three Cd solutions, respectively. The exposure started after the end of egg laying (indicated by “day 0”) for a duration of 24 hours (until day 1, Fig. 1). After this period of exposure, eggs were transferred to metal-free conditions (demineralized water) for the rest of the embryonic development.

Three sampling dates were considered: day 1, corresponding to the end of exposure (control or one of the three Cd concentrations), and defining the beginning of the embryonic development; day 6, marking the start of organogenesis; and day 12, delimiting its completion (see states of development in Fig. 1A).

Three exposure regimes were carried out (Fig. 1A), in which a total number of ten different clutches were consumed. Three of them were used for Cd measurement in eggs (regime I). Four other clutches were applied for PCR and qRT-PCR analyses (regime II). The last three clutches were used for hatching success determination (regime III). Eggs of a same clutch were tested exclusively in one of the three exposure regimes, but proportionally subjected to one of the four treatments (control and three Cd concentrations) within the same exposure regime.

### Exposure regime I and sample processing for Cd analysis

In the exposure regime I, three separate clutches were used for the determination of Cd concentrations in the entire egg (consisting of eggshell, albumen and embryo) (Fig. 1A).

At four sampling dates (days 0, 1, 6 and 12) five eggs (approximately 30 mg dry mass) were pooled from each of the four exposure treatments (Fig. 1A and 1B). Day 0 was considered as the first sampling point in order to assess Cd levels in freshly laid eggs prior to exposure.

Pooled samples were oven-dried (50°C) and subsequently digested in 300 μL of nitric acid (HNO_3_ 67–69%, quality Optima for ultra trace analysis, Fisher Scientific) for 40 h at 60°C. After digestion, ultrapure water was added to a final volume of 15 mL. Cd concentrations in the samples were measured using ICP-MS (X series II, Thermo scientific, France). The reliability of the measurement procedure was assessed by applying different types of certified standard reference materials such as dogfish liver (DOLT) and lobster hepatopancreas (TORT) (both: NRCC-CNRL, Canada).

### Exposure regime II and sample preparation for PCR and qRT-PCR

Exposure regime II was carried out in order to detect the transcription of the *MT* isoform genes through embryonic development by applying PCR and qRT-PCR on eggs from four clutches (Fig. 1B).

For PCR of the three *MT* isoform gene transcripts, snail egg samples were used at day 1 (control, and 5, 10, 15 mg/L Cd-exposed), day 6 (control and 5mg/L Cd exposed) and day 12 (control only), (Fig. 1). Since 1-day-old embryos were very small (diameter < 500 μM), 15 of them were pooled for each of the exposure treatments applied. Three embryos were pooled to one sample for 6 and 12-day-old individuals (Fig. 1B).

The small amount of tissue of 1 day-old embryos did not permit to apply qRT-PCR to these samples. Consequently, only eggs from the two successive sampling dates (6 and 12 days) were used for the quantitative approach. At these two dates, three embryos from each treatment control and Cd-exposed eggs were separated from albumen (Fig. 1B) and processed for RNA isolation as described below. Pooled samples were soaked in RNAlater (Applied Biosystems, Foster City, CA, USA) and stored at -80°C. RNA isolation was performed using the RNeasy Plus Micro Kit and the RNeasy Mini Kit (Qiagen, Hilden, Germany) according to the manufacturer’s instructions. The Quant-iT RiboGreen RNA Assay Kit (Invitrogen, Karlsruhe, Germany) was used for RNA quantification after DNase I (Fermentas, St. Leon-Rot, Germany) digestion. 250 ng of RNA were applied in a 50 μL approach for cDNA synthesis using the RevertAid H Minus M-MLV Reverse Transcriptase kit (Fermentas).

### PCR and qRT-PCR of the three MT isoforms

The presence of *MT* isoform gene transcripts was investigated by means of PCR in 1 day, 6 day and 12-day-old embryos. The isoform gene-specific primers were designed for *C*. *aspersus*, and the amplified fragments sequenced according to Höckner et al. [13]. The primers were applied as follows: for CdMT sense, 5’-GCC GCC TGT AAG ACT TGC A-3’ and antisense, 5’-CAC GCC TTG CCA CAC TTG-3’; for CuMT: sense, 5’-AAC AGC AAC CCT TGC AAC TGT-3’ and antisense: 5’-CGA GCA CTG CAT TGA TCA CAA-3’; for Cd/CuMT: sense, 5’-TGT GGA GCC GGC TGT TCT-3’ and antisense: 5’-CAG GTG TCA TTG TTG CAT TGG-3’. Amplifications of the three different *MT* isoforms were performed with 1 μL (for 6 and 12 day samples) or 2 μL (for 1 day samples) of cDNA template, 0.5 μL of gene-specific sense and antisense primer (10 μM), 0.5 μL of dNTPs (10 mM) and 2.5 μL of 10X Taq Buffer), using 0.5 μL of Titanium Taq DNA Polymerase (Clontech) to a final volume of 25 μL. The cycling parameters were as follows: one initial denaturation step of 2 min at 95°C followed by 30 (or 35 for 1 day) cycles at 95°C for 20 s, 54.3 (*Cd/CuMT*), 57.2 (*CdMT*) or 58.5°C (*CuMT*) for 25 s, 68°C for 30 s and a final extension of 10 min at 70°C. Amplification products were visualized on 3% agarose gels stained with GelRe (Biotum, Hayward, CA, USA) after an electrophoresis conducted at 110 V for 45 min. The amplicon lengths of the single *MT* isoform gene transcripts were 56 bp (*CdMT*), 74 bp (*CuMT*) and 59 bp (*Cd/CuMT*), respectively.

qRT-PCR was carried out according to the protocol reported by Höckner et al. [13]. Briefly, 2 μL of sample cDNA were added to 2 μL of BSA 10X (Sigma-Aldrich, USA), 2 μL of sense primer, 2 μL of antisense primer, 2 μL of ultrapure water and 10 μL of Power SYBRGreen (Applied Biosystems, Foster City, CA, USA) for a final volume of 20 μL. The following primer concentrations were used: CdMT sense, 300 nM; CdMT antisense, 900 nM; CuMT sense, 900 nM; CuMT antisense, 900 nM and Cd/CuMT sense, 900 nM; CdCu/MT antisense, 300 nM. The analyses were performed using a 7500 Real Time PCR analyzer (Applied Biosystems). For each cDNA sample, qRT-PCR measurements were performed in triplicates. The efficiency of qRT-PCR reactions was between 95 and 99%. The relation between resulting Ct values and the log of copy numbers of each gene were for *CdMT*, Ct = - 3.4466 log copy + 34.987; for *CuMT*, Ct = - 3.3178 log copy + 34.483; and for *Cd/CuMT*, Ct = - 3.3385 log copy + 32.873.

The obtained values expressed in copy numbers were normalized using total RNA in samples and were given in copy numbers / 10 ng of total RNA.

### Exposure regime III and determination of hatching success

In exposure regime III, the three egg clutches were used for determination of Cd toxicity parameters (Fig. 1A). Each clutch was separated into four groups corresponding to one of the four treatements: control (demineralized water) and three Cd concentrations (Fig. 1B). After 24 hours, eggs were placed on filter paper with demineralized water for the remainder of the developmental period. Twenty days after the beginning of the experiment, the average hatching success for each concentration was calculated (Fig. 1A). The results were considered valid if the hatching success of controls was at least 70% of the average value normally observed for controls under laboratory rearing conditions.

### Statistics

All statistical analyses were performed with the R software (R Development Core Team, 2004, http://www.R-project.org/; version 2.13.2). The homogeneity and normality of data were tested using the Bartlett test and the Kolmogorov-Smirnov test, respectively. Since the data were not normally distributed, non-parametric tests were used for all statistical analyses. The Mann-Whitney *U* test was used to compare i) Cd accumulation in eggs for each solution (i.e., control versus 5 mg/L, 5 mg/L versus 10 mg/L, etc.) and ii) qRT-PCR results in control and Cd-exposed samples at 6 and 12 days. Kruskal-Wallis test was used to compare i) levels of Cd measured in eggs at each date for the same concentration of exposure (i.e., at 1, 6 and 12 days for 5 mg/L exposure), and ii) differences between the 3 *MT* gene transcript values of controls (comparisons of *CdMT*, *CuMT* and *Cd/CuMT* values at 6 and 12 days in control). In all cases, statistical significance was set at p < 0.05. The dose-dependent curves and the EC_10_ and EC_50_ values were determined with the Hill’s model using the macro application Regtox free version EV6.1 in Excel.

## Results

### Cd concentration in eggs

Cd concentrations in eggs increased significantly in a dose-dependent manner (Cd in 15 mg/L-exposed eggs> 10 mg/L> 5mg/L) (Table 1): Cd accumulation in eggs exposed to 15 mg/L were significantly higher than those exposed to 10 mg/L (W = 78, *p* < 0.001) or 5 mg/L (W = 0, *p* < 0.001). Cd concentrations in eggs exposed to 10 mg/L were higher than those exposed to 5 mg/L (W = 81, *p* < 0.001). No significant change of Cd concentrations was found in embryos through development time (from day 1 to 12) for any of the Cd concentrations applied: instead, Cd concentrations remained near 12.5 μg/g DW (KW = 0.71, df = 3, *p* = 0.87), 23 μg/g DW (KW = 0.62, df = 2, *p* = 0.73), and 40 μg/g DW (KW = 3.84, df = 3, *p* = 0.28) for eggs exposed to 5, 10 and 15 mg Cd/L, respectively (Table 1).

**Table 1:** Cadmium concentrations (mean and standard deviations in brackets) in eggs of *Cantareus aspersus* recorded throughout the duration of the experiment (0–12 days).

Days of measurement		0	1	6	12
	Ctrl	**0.01** ^**a**^ **(+/- 0.01)**	**0.08** ^**a**^ **(+/- 0.13)**	**0.11** ^**a**^ **(+/- 0.13)**	**0.01** ^**a**^ **(+/- 0.01)**
**Cd concentration (μg/g dry weight)**	5mg/L	—	**14.18** ^**b**^ **(+/- 3.70)**	**12.67** ^**b**^ **(+/- 1.32)**	**13.25** ^**b**^ **(+/- 1.36)**
	10mg/L	—	**23.17** ^**c**^ **(+/- 3.69)**	**23.32** ^**c**^ **(+/- 3.57)**	**23.29** ^**c**^ **(+/- 4.99)**
	15mg/L	—	**37.99** ^**d**^ **(+/- 11.73)**	**37.04** ^**d**^ **(+/- 6.86)**	**35.63** ^**d**^ **(+/- 4.97)**

For each point of measurement, pooled samples from three replicate exposures were considered (*n* = 3), showing means and standard deviations (in brackets). Significant differences of values between single treatments and times were expressed by different superscript lower case letters (a, b, c, d) (Mann-Whitney *U* test, *p* < 0.001).

### Hatching success of snail eggs

The EC_10_ and EC_50_ values based on hatching success were 2.68 and 5.05 mg Cd/L, with confidence intervals (CI) of 95% from 2.05 to 3.32 and 4.41 to 5.79, respectively.

### Expression of the three *MT* isoform genes

Due to the low quantity of RNA available from pools of 15 one-day-old embryos, the four pools per treatment were merged to obtain one major pool of 60 embryos for each of the four exposure concentrations. Hence, the PCR on one-day-old samples was performed on these 4 major pools of 60 embryos each. Yet, the small amount of tissue in these major pools did not permit to apply qRT-PCR.

**Fig 2:**
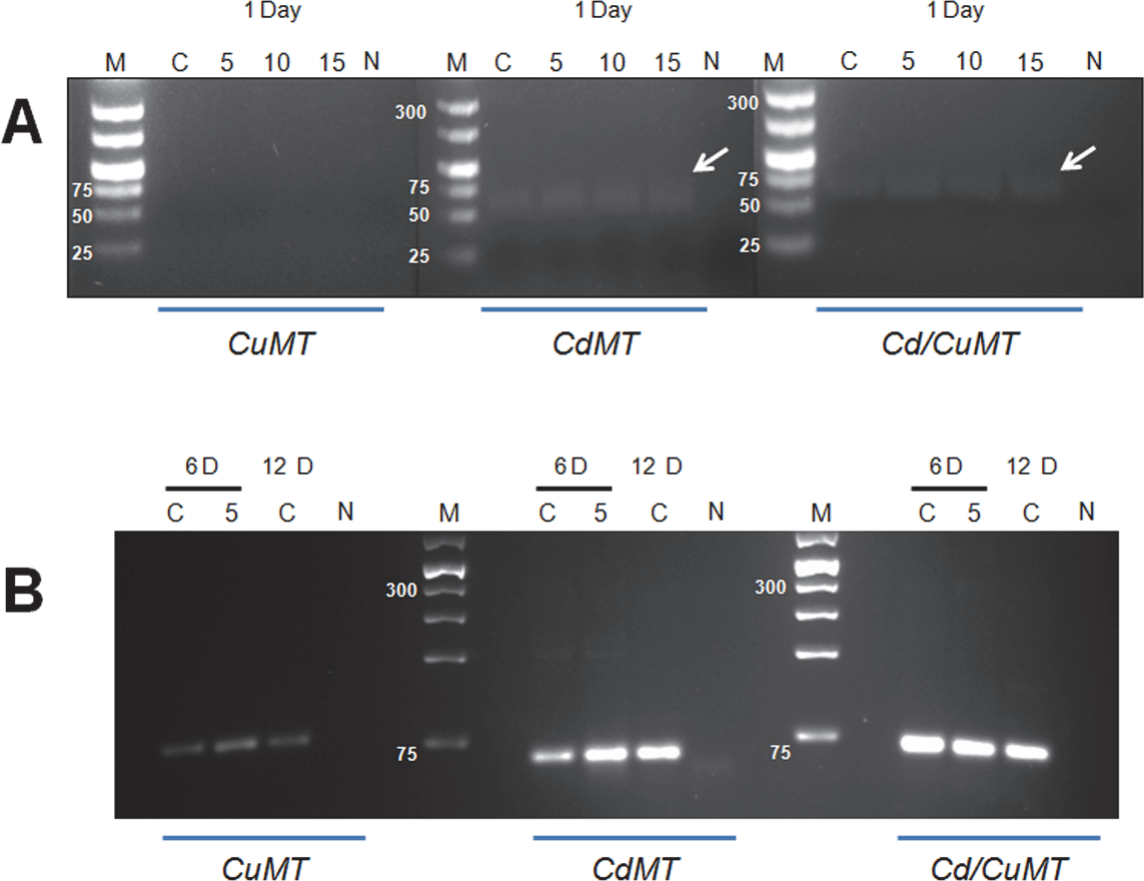
Visualization of PCR amplification bands specific for the three *MT* isoform genes (*CuMT*, *CdMT* and *Cd/CuMT*) of *Cantareus aspersus* control and Cd-exposed embryos. (A) Detection of *MT* isoform gene amplification bands in 1-day-old control eggs (C) and embryos exposed for one day to increasing Cd concentrations (5, 10, and 15 mg/L) (5, 10, 15), along with DNA ladder bands (M) (Generuler Low range DNA ladder and 100pb plus DNA ladder, Thermo Scientific) on 3% agarose gel. N, negative control without sample DNA. (B) Detection of *MT* isoform gene amplification bands in 6-day-old control (C) embryos and embryos exposed to 5 mg Cd/L (5), or in 12-day-old embryos (C, controls only), along with DNA ladder bands (M) (Generuler Low range DNA ladder and 100pb plus DNA ladder, Thermo Scientific) on 3% agarose gel. N, negative control without sample DNA.

Consequently, the expression of the *CdMT* and *Cd/CuMT* genes, but not that of the *CuMT* gene, could be detected in 1-day-old embryos (exposed or not) by PCR (Fig. 2A). Only at the age of 6 days and thereafter were the three *MT* isoform genes altogether expressed in snail embryos (Fig. 2B). However, the intensity of PCR bands for the *CuMT* gene remained the lowest one in 6 and 12-day-old control samples compared to the bands for the other two *MT* genes. In contrast, the PCR band with the highest intensity throughout appeared to be that of the *Cd/CuMT* gene (Fig. 2B).

**Fig 3:**
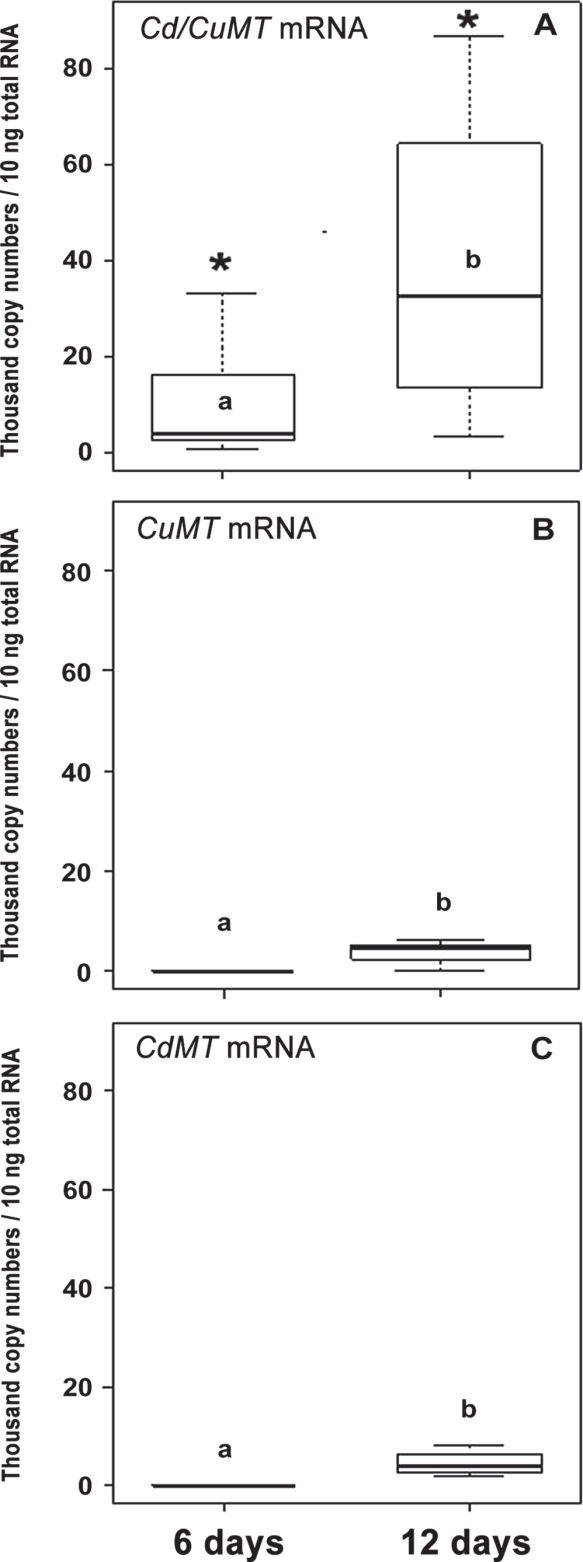
*MT* isoform mRNA transcription values (thousand mRNA copy numbers/10 ng of total RNA) for the *Cd/CuMT* (A), *CuMT* (B) and *CdMT* (C) genes of *Cantareus aspersus* (*n* = 4) in control (unexposed) eggs through embryonic development (6 and 12 days). Box plots indicate the median values (bold line) for each group, the edges of the boxes are the 25^th^ and 75^th^ percentiles. The whiskers represent the 10^th^ and 90^th^ percentiles (*). Significant differences (Kruskal-Wallis test, p < 0.03) between *Cd/CuMT*, *CdMT* and *CuMT* values at 6 and 12 days. Differing lower case letters (a, b) indicate significant differences between single values for each of the three *MT* isoform genes at days 6 and 12 (Mann–Whitney *U* test, *p* < 0.02).

As shown by qRT-PCR, constitutive transcription levels of all three *MT* isoform genes increased with development time (6 and 12 days) in unexposed snail embryos (W = 0, *p* < 0.05, between days 6 and 12) (Fig. 3). Over all two dates investigated (6 and 12 days), the transcription values of the *Cd/CuMT* gene was significantly higher than those of the other two *MT* genes (KW = 8.87; 7, df = 2, *p* = 0.01; 0.03 at 6 and 12 days, respectively) (Fig. 3).

**Fig 4:**
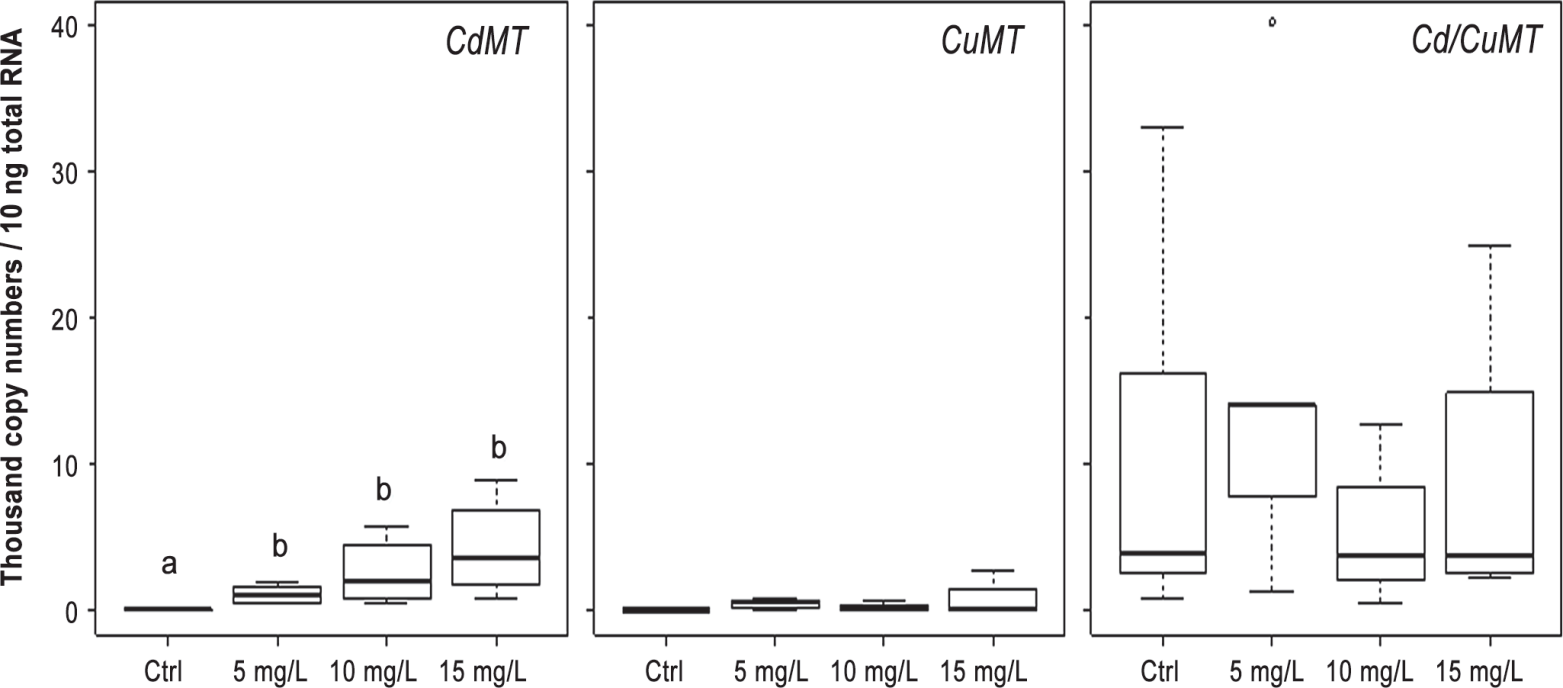
*MT* isoform mRNA transcription values (thousand mRNA copy numbers/10 ng of total RNA) of the *CdMT*, *Cd/CuMT* and *CuMT* genes in 6-day-old embryos of *Cantareus aspersus* (*n* = 4) exposed to increasing Cd concentrations (C, control; 5 mg/L, 10 mg/L and 15 mg/L). Box plots indicate the median values (bold line) for each group, the edges of the boxes are the 25^th^ and 75^th^ percentiles. The whiskers represent the 10^th^ and 90^th^ percentiles. The white dot is the extreme value. Significant differences (Mann-Whitney *U* test, *p* < 0.005) of transcription levels for the three *MT* isoform genes between control and exposed embryos are indicated by differing lower case letters (a, b).

**Fig 5:**
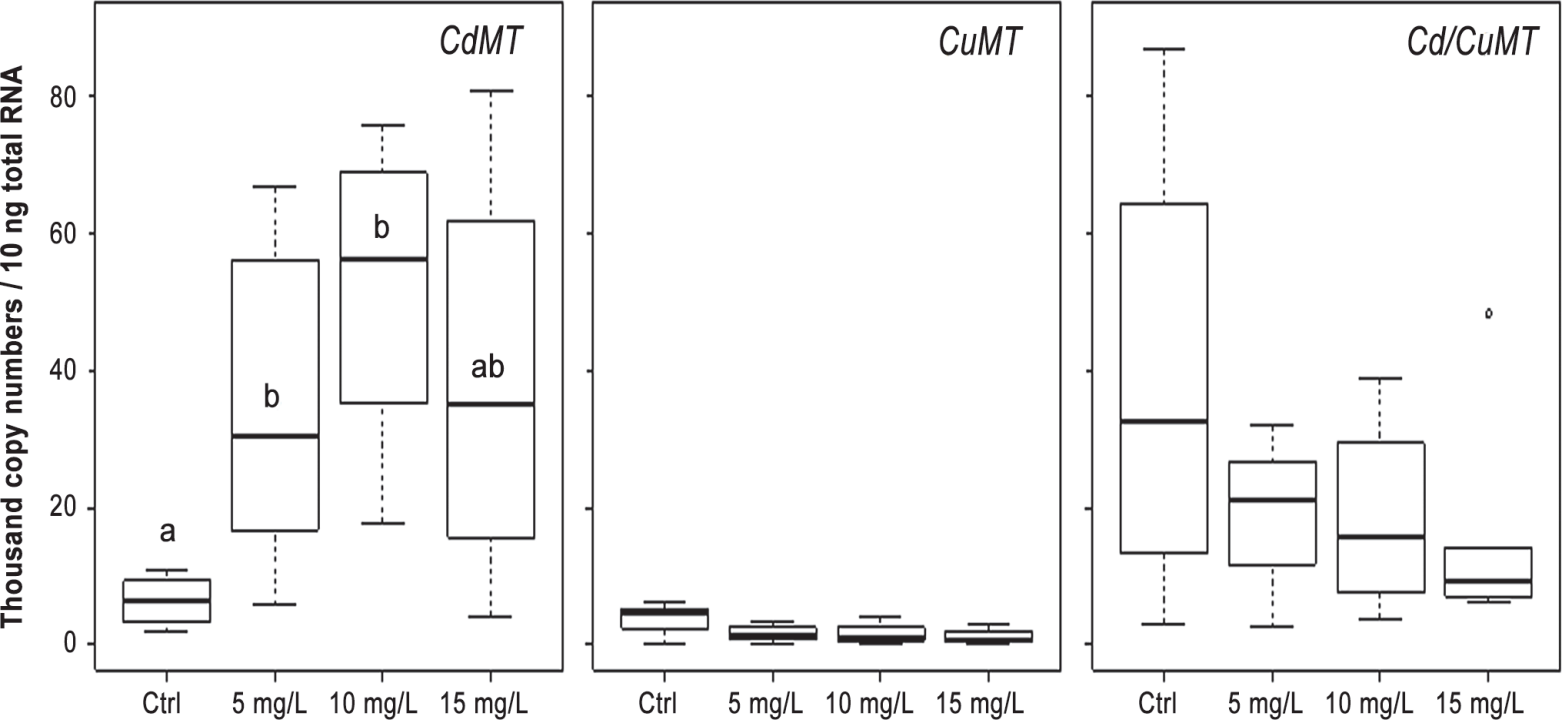
*MT* isoform mRNA transcription values (thousand mRNA copy numbers/10 ng of total RNA) of the *CdMT*, *Cd/CuMT and CuMT* genes in 12-day-old embryos of *Cantareus aspersus* (*n* = 4) exposed to increasing Cd concentrations (C, control; 5 mg/L, 10 mg/L and 15 mg/L). Box plots indicate the median values (bold line) for each group, the edges of the boxes are the 25^th^ and 75^th^ percentiles. The whiskers represent the 10^th^ and 90^th^ percentiles. The white dot is the extreme values. Significant differences (Mann-Whitney *U* test, *p* < 0.05) of transcription levels of the three *MT* isoform genes are indicated by differing lower case letters (a, b).

In 6-day-old embryos exposed to Cd, a significant induction (W = 0, *p* = 0.02 for each concentration) of transcription levels was detected for *CdMT* mRNA, in contrast to the non- significant responses of the *CuMT* (*p* > 0.11) and the *Cd/CuMT* genes (*p* > 0.69) (Fig. 4). This Cd-specific transcriptional induction of the *CdMT* gene was also observed in 12-day-old embryos (Fig. 5) exposed to 5 and 10 mg Cd/L (W = 2, *p* = 0.05; W = 0, *p* = 0.01, respectively). In contrast to the *CdMT* gene, the transcription rates of the *CuMT* and *Cd/CuMT* gene in 12-day-old embryos were not significantly induced by Cd at all (*p* > 0.40 and *p* > 0.34) (Fig. 5).

## Discussion

### Expression pattern of *MT* isoform genes in unexposed embryos

In the present study, data on the expression pattern of the *CdMT*, *CuMT* and *Cd/CuMT* isoform genes in the ELS of the terrestrial snail *Cantareus aspersus* are shown for the first time. It appears that snail embryos are able to transcribe the *CdMT* and *Cd/CuMT* genes from the first 24 hours of their development, corresponding to the beginning of the morula stage [23]. At the same time of development, no indication for an early expression of the *CuMT* gene was observed (Fig. 2A, B). Our results are partially in accordance with studies by Russo et al. [27,28] in the sea urchin *Paracentrotus lividus*, where a low expression rate of *MT* mRNA in eggs or embryos at the eight cells and morula stages was reported. On the other hand, the absence of *CuMT* gene expression in *C*. *aspersus* morula embryos indicates that at this early stage of development the cell-specific homeostatic regulation of Cu which is linked to the expression of *CuMT* in mollusk rhogocytes [11] may not be differentiated yet. Only from day 6 of embryonic development could the expression of the three snail *MT* isoform genes (*CdMT*, *CuMT* and *Cd/CuMT*) be observed altogether (Fig. 3), indicating that from this point of development, the metal regulation and detoxification capacities in *C*. *aspersus* embryos may be fully unfolded.

In fact, the stepwise increase of transcription levels of the three *MT* isoform genes from 6 to 12 days of development may be related to gradual tissue differentiation within this time (Fig. 3). Six-day-old snail embryos start their organogenesis by development of a primary foot, mouth, and anus, whereas after 12 days, embryos assume the appearance of small adult snails with the presence of all distinct parts of the body (shell, eyes, mantle, digestive glands, etc.) [23]. In our experiments, twelve-day-old embryos were clearly more efficient in the expression of the three MT genes compared to earlier developmental stages (Fig. 3). Russo et al. [28] attributed high levels of gene expression at the onset of the gastrulation stage of the sea urchin *P*. *lividus* to an increased requirement of *MT* mRNA due to the organization of new tissue territories. In *C*. *aspersus*, the gradual rise of *MT* isoform gene transcription over time of development of unexposed embryos proves the significance of constitutive *MT* gene expression, suggesting an important role of the three *MT* isoforms during the snail embryogenesis. For the very first stages of sea urchin embryogenesis, Russo et al. [28] suggested a possible role of maternal *MT* mRNA supplies during oogenesis, giving rise to the early ability of *MT* expression in sea urchin embryos. Such a maternal *MT* mRNA stock might be used by the embryos for the purpose of metal homeostasis during early development when a high level of essential metals is needed to cope with the requirements of rapid DNA synthesis and metabolic activity. In eggs of the marine mollusk *Crassostrea virginica*, Roesijadi et al. [29] have also observed the presence of maternal *MT* mRNA, which declines immediately after fertilization. In the case of *C*. *aspersus* embryogenesis too, a role of maternal *MT* mRNA during early development cannot be excluded, although the absence of *CuMT* mRNA transcription in one-day-old embryos does not support this hypothesis. Rather, the gradual increase of *MT* gene expression from six to twelve-day-old embryos can most likely be attributed to the rising ability of developing snails to transcribe the three *MT* genes constitutively by their own (Fig. 3). This suggests that the physiological capacity of metal regulation and detoxification may gradually increase throughout the snail embryogenesis, as similarly reported by Lemoine et al. [30] for the marine mollusk *Mytilus edulis*.

Overall, we believe that the high standard deviations of values obtained in our qRT-PCR measurements (Fig. 3) may reflect the high biological variability of individual embryos and/or pooled embryo samples due to the fact that they refer to the whole body of very small-sized developing organisms. In this concern, we would like to remind that similarly high variabilities of copy numbers of *MT* isoform genes from terrestrial snails were normally also encountered for adult in our previous measurements [10, 13, 19].

In unexposed embryos of *C*. *aspersus*, the highest transcription rate among all three *MT* isoform mRNAs was that of the *Cd/CuMT* gene. This is interesting, since in adult snails, this same *MT* isoform gene is normally expressed at a much lower rate [13]. This suggests the marginal importance of this isoform gene in metal balance of adult snails, contrasting with its apparently more prominent physiological importance in developing snail embryos, perhaps in accordance with their increased metal homeostatic and metabolic requirements during development. Russo et al. [28] suggested such a homeostatic role for MTs in sea urchin embryos too.

The involvement of *MT* genes and proteins during development has also been suggested for several vertebrate species. Olsson et al. [31], for example, have suggested an important role of *MT* genes during development and differentiation in the ELS of rainbow trout (*Salmo gairdneri*). Moreover, a significant function has also been attributed to MTs in the embryonic brains of the amphibian *Xenopus laevis* [32] and the lizard *Podarcis sicula* [33]. The increase of *MT* mRNA in lizard embryos at hatching was attributed to the fact that at this point the developing organism switches from a closed system to an open system physiology owing to the loss of eggshell protection.

### Up-regulation of *MT* genes in cd-exposed embryos

The present study shows that during exposure of snail embryos to Cd over 24 hours, the metal is taken up from the exposure solution by the entire egg (shell, albumen and embryos). In fact, Cd concentrations in eggs of *Cantareus aspersus* after this short-term exposure remained elevated and stable throughout the 12 days of the experiment (Table 1). On the other hand, Cd concentrations in eggs of the present study revealed a dose-dependent metal uptake (Table 1). Cd concentrations measured in one-day-old eggs most likely represent the metal load present in the eggshell. According to the distribution of Cd in eggshell and albumen after 7 days of exposure reported by Druart et al. [23], in 6 and 12-day-old eggs, however, Cd concentrations analyzed may possibly reveal the metal content of the albumen. This may be due to the time required by Cd to diffuse throughout the different parts of the egg, thereby exerting toxic effects on snail embryos. The significant up-regulation of the *CdMT* gene transcription at 6 days of development provides evidence, in any case, that the Cd has by this time reached the embryonic tissue, which responds to the metal insult by *CdMT* gene expression.

Overall, this study provides the first data on the expression and up-regulation of *MT* genes in the ELS of a terrestrial snail (*C*. *aspersus*) exposed to Cd (Figs. 4 and 5). In spite of the high standard deviations of qRT-PCR values obtained (see above), a dose-dependent up-regulation of the *CdMT* gene was found, in both, six-day-old and 12-day-old exposed embryos. The only exception was seen in 12-day-old embryos at the highest Cd exposure concentration, where the *CdMT* mRNA transcription level did not increase any more compared to lower exposure concentrations (Fig. 5). A rapid induction of *MT* expression was also reported by Roesijadi et al. [29] for Cd-exposed embryos of *C*. *virginica*. However, the *MT* gene induction in oyster eggs occurred at a faster rate compared to that observed in the snail eggs of the present study, likely because oyster eggs were directly exposed to the metal through the contaminated seawater and thus experienced a faster Cd transfer into the embryonic cells.

Whereas Roesijadi et al. [34] have associated the increased *MT* gene expression of *C*. *virginica* in response to Cd exposure with the ability of this species to protect itself from metal toxicity, Meistertzheim et al. [35] have hypothesized that in the Pacific oyster (*C*. *gigas*), high MT levels may enable embryos of this species to survive in stressful environments. In many species, however, exposure to toxic metals directly leads to an increase of *MT* gene transcription [30]. In the present study too, the increase in transcription rates of the Cd-specific *MT* isoform gene upon exposure to this metal demonstrates the ability of premature (6-day-old) snail embryos to immediately respond to the Cd stress by synthesizing a metal-specific MT isoform able to inactivate the metal by detoxification. The fact that in 12-day-old embryos exposed to the highest Cd concentration (15 mg/L) the mRNA copy numbers of the *CdMT* gene did not exceed the respective transcription rates observed at lower exposure concentrations (5 and 10 mg Cd/L) (see Fig. 5) perhaps indicates that at this highest exposure concentration, Cd may start to exert toxic effects which cannot be compensated any more by *CdMT* expression. Indeed, our results show that at an exposure concentration of 15 mg Cd/L, the hatching success (at 20 days) of snail embryos is completely inhibited. Therefore, our interpretation is that at this high Cd concentration, embryos lost their ability to further up-regulate the Cd-specific *MT* gene and hence, their defense capacities became overloaded. Chabicowski et al. [36] have shown that in adult Roman snails (*Helix pomatia*) exposed to increasing Cd concentrations CdMT overloading is accompanied with an increase of individual mortality. Interestingly, Roesijadi et al. [37] also observed, in a similar manner as in the present study, a decrease of *MT* mRNA inducibility in hemocytes of *C*. *virginica* exposed to Cd in *vitro* above a threshold concentration of 35 μM.

In the present study, the expression pattern of the three *MT* isoform genes in Cd-exposed embryos of *C*. *aspersus* is similar to that reported earlier by Höckner et al. (2011) [13] for adult snails. In both cases, Cd-specific induction was only seen for the *CdMT* gene, whereas no metal-responsive increase of transcription rates was observed for the *CuMT* and the *Cd/CuMT* genes (see Figs. 4 and 5). Whilst the lacking inducibility of the *CuMT* gene by Cd exposure may be understandable because of its exclusive Cu specificity [19], the inability of the *Cd/CuMT* gene to respond at the transcriptional level to Cd stress is more surprising. In fact, the expressed Cd/CuMT isoform is able to bind both Cd^2+^ and Cu^+^ ions simultaneously upon metal exposure [12]. Hence, it has to be assumed that in spite of the lacking metal-dependent inducibility of this gene, its constitutive expression in snail embryos may be sufficient to make it available as a Cu^+^ and Cd^2+^ binding ligand for purposes of metal regulation and detoxification, respectively. At least, our data suggest that this *MT* isoform gene must play, due to its elevated transcription rate, a so far unknown, but perhaps important role in snail embryogenesis.

## Conclusions

The present study reveals the early capacity of developing embryos of the terrestrial snail *Cantareus aspersus* to: (i) constitutively express the three characteristic *MT* isoform genes (*CuMT*, *CdMT* and *Cd/CuMT*) with (ii) a higher transcription level for the unspecific *Cd/CuMT* gene compared to that known from adult snails; and (iii) a Cd-specific response of the *CdMT* gene by increased transcription rates upon metal exposure during embryogenesis. Because of this, the ELS of *C*. *aspersus* may be used in ecotoxicological studies, e.g. by quantification of the *CdMT* gene expression as a biomarker of Cd exposure. As to the high constitutive expression level of the *Cd/CuMT* isoform gene, a possible role of this gene during embryogenesis must be considered. Overall, the present findings may constitute a first step towards better understanding the high Cd tolerance of the ELS in this terrestrial snail species.
